# How frequent are non-evidence-based health care beliefs in chiropractic students and do they vary across the pre-professional educational years

**DOI:** 10.1186/s12998-018-0178-y

**Published:** 2018-03-15

**Authors:** Stanley I. Innes, Charlotte Leboeuf-Yde, Bruce F. Walker

**Affiliations:** 1 School of Health Professions, Murdoch University, Murdoch, Australia; 2 Institut Franco-Européen de Chiropraxie, 94200 Ivry sur Seine, France; 3 CIAMS, University Paris-Sud, Université Paris-Saclay, 91405 Orsay Cedex, France; 4 CIAMS, Université d’Orléans, 45067 Orléans, France; 5 Institute for Regional Health Research, University of Southern Denmark, DK-5000 Odense, Denmark

**Keywords:** Chiropractic, Education, Evidence-based, Beliefs, Scope of practice

## Abstract

**Background:**

Evidence suggests that a students’ beliefs already prior to entering a program may be important as a determinant in sustaining unsuitable health care beliefs. Our objectives were to investigate the proportion of Australian chiropractic students who hold non-evidence-based beliefs in the first year of study and the extent to which they may be involved in non-musculoskeletal health conditions. Finally, to see if this proportion varies over the course of the chiropractic program.

**Method:**

In 2016, students from two Australian chiropractic programs answered a questionnaire on how often they would give advice on five common health conditions in their future practices as well as their opinion on whether chiropractic spinal adjustments could prevent or help seven health-related conditions.

**Results:**

From a possible 831 students, 444 responded (53%). Students were highly likely to offer advice (often/quite often) on a range of non-musculoskeletal conditions. The proportions were lowest in first year and highest the final year. Also, high numbers of students held non-evidence-based beliefs about ‘chiropractic spinal adjustments’ which tended to occur in gradually decreasing in numbers in sequential years, except for fifth year when a reversal of the pattern occurred.

**Conclusions:**

New strategies are required for chiropractic educators if they are to produce graduates who understand and deliver evidence-based health care and able to be part of the mainstream health care system.

**Electronic supplementary material:**

The online version of this article (10.1186/s12998-018-0178-y) contains supplementary material, which is available to authorized users.

## Background

Chiropractic educational regulatory agencies uniformly aim to produce graduates capable of best practice care and interdisciplinary collaboration [1]. Arguments have also been voiced for chiropractors to become known as non-surgical spinal care experts [2]. However, studies have shown the existence of aberrant chiropractic practice profiles which include anti-vaccination beliefs and excessive X-ray usage [3]. Further these chiropractic practices consider ‘wellness care’ to be a main component of practice and treat a high number of asymptomatic patients for somato-visceral conditions [3, 4]. This type of profile is considered ‘unsuitable’ within the context of contemporary evidence-based health care [5] and, not surprisingly, chiropractors with profiles like this were less likely to receive referrals from or make referrals to medical practitioners than those whose main sector of activity was more musculoskeletal based with profiles such as treating sports injuries and prescribing exercises [4].

These unsuitable practice profiles are based on beliefs which are not biologically plausible nor are they supported by available evidence. Some specific examples of this are beliefs that spinal manipulation can influence the immune system, improve Attention Deficit Disorder or somato-visceral conditions [6]. It is logical to assume that, since all chiropractors were once students, education may play a role in this and the logic is not without evidence. One study has found that unsuitable views in chiropractic practitioners can be traced back to their college of education [7]. To further this proposition, evidence of these unsuitable beliefs has been shown in chiropractic students. A survey of one North American chiropractic program [8] revealed that 11% of students agreed or strongly agreed that all disease is caused by ‘vertebral subluxation complexes’ and that chiropractic spinal adjustments are an effective primary treatment for AIDS (9%), cancer (12%), depression (44%), inner ear infections (59%), and asthma (61%). Finally, 80% of the students in that study believed that all patients should have lifetime chiropractic care. Clearly this profile is not in the best interests of patient safety, quality care, or public health.

However, 3 in every 4 of these chiropractic students were found to rate evidence as more important than traditional chiropractic theory (vertebral subluxation complexes) [8]. If evidence is the most important determinant, how is it possible to have non-evidence based or unfounded beliefs that appear biologically implausible? This suggests that either students may not be taught how to recognize and understand the evidence or are refusing to integrate evidence which is at odds with their perceived professional identity. Other explanations may also be possible.

It is unknown if these unfounded beliefs are the same internationally. At first glance it would appear not. A study comparing Australian health care students in the final year of chiropractic, medicine, physiotherapy / physical therapy, occupational therapy and pharmacy found that the chiropractic students were most likely to offer guideline-consistent recommendations as well as possess the most helpful beliefs about LBP and impairment [9].

These international variations could perhaps be explained by differing cultures or educational standards. This is plausible, as we have previously found variations between the accreditation standards of the regulatory bodies who oversee the chiropractic programs in the USA, Australia, Europe and Canada [1, 10]. Research confirms the phenomenon that it is possible for students to hold views, like those cited above, but still offer guideline-based care [11]. This implies that chiropractic students, even if they are prepared to offer guideline based care, may also hold unreasonable beliefs in other areas. However this specific possibility has not been studied.

The aim of this research is to perform an exploratory study into Australian chiropractic students’ views on various aspects of chiropractic practice that appear lacking plausibility and/or evidence. Specifically, we were interested in the following questions:

1. Do Australian chiropractic students believe that upon graduation they should involve themselves in the management of non-musculoskeletal disorders?
2. Do Australian chiropractic students believe chiropractic treatment will have preventive effects on various conditions?
3. If there is a proportion of students who hold these beliefs (1–3), does the proportion vary over the 5 years of the chiropractic program?

## Methods

### Study procedure

A cross-sectional study was carried out. The entire student population at two chiropractic programs within Australian Universities (Murdoch and Macquarie universities) were used for data collection between October and November in 2016. An anonymous class-room handout questionnaire was chosen as the instrument of measurement, as it facilitated the collection of a large amount of robust data in a timely and cost-effective manner.

A team consisting of the three authors were responsible for the design of the questionnaire and four 4th year chiropractic students from Murdoch University assisted with the survey administration and data collection. Recruitment, data administration, and collection of questionnaires were provided by administrative staff at the two universities.

Ethics approval was granted by Murdoch University Human Research Ethics Committee (Project No 2016/118).

### The questionnaire

The survey contained four sections and was too large to present in one study. The results of some sections have been reported elsewhere [12]. For this study two of these sections were used (Additional file 1).

The first section sought demographic details (chiropractic program, sex, year of study). The second section had two sets of questions in order to address our objectives on the prevalence of non-evidence-based beliefs in chiropractic students (Additional file 1).

The first set of questions in this section asked students how often they would give advice to patients in their practices for five common health conditions: stress, cardiovascular disease, diabetes, musculoskeletal (MSK) problems, and wellness in general. Their response options were “No or rarely”, “Sometimes” or “Quite often or often”. Some of these health conditions were within the scope of chiropractic practice (e.g. musculoskeletal problems) whilst others were not (e.g. diabetes). Therefore, it would be considered unsuitable to provide frequent advice on the former conditions rather than on the latter. This was to gain an insight into students’ understanding of chiropractic care, as indicated by how often they would provide advice on matters inside and outside their scope of practice. It was assumed that chiropractic students would be more likely to offer advice on musculoskeletal conditions as this is the major component of the curriculum. Other health conditions are taught to inform diagnosis and appropriate management of musculoskeletal conditions. For example, people who have diabetes may have delayed recovery times. The training does not encompass the full range of clinical tests for diagnosis and appropriate pharmacological management. Appropriate diet [13] and exercise [14] have been shown to have therapeutic benefits and could be reasonably included in an initial assessment and on-going monitoring by a chiropractor. However full dietary assessment, monitoring of blood sugar levels and appropriate insulin dosage would not be and should rather be met by a number of specialist resources and practitioners available to people with diabetes in Australia. Consequently we would expect students responses to be more frequently “no or rarely” or “sometimes” to the question on non-musculoskeletal conditions.

The second set of questions in this section asked students for their opinion as to whether ‘chiropractic spinal adjustments (CSA)’ could prevent or help seven health-related conditions. These conditions were selected by the authors from previous research reports, which had identified them as being indicative of unsuitable practice profiles [3, 4, 7]. These health issues were biologically implausible, without any supportive evidence and therefore outside the scope of chiropractic practice, such as “chiropractic spinal adjustments can prevent disease in general [15–17]”, “… help the immune system [18]”, “… make it easier to give birth [19–21] and improve the health of infants [22–25]”. The other conditions were related specifically to spinal care and also were contrary to, or without supportive evidence [24, 26–28]. These included, for example: “can chiropractic spinal adjustments prevent degeneration of the spine and chronic back pain?” The response options were “Definitely not”, “Probably not”, “I don’t know”, “Yes, probably” and “Yes, definitely”. We considered the two last options as unsuitable.

### Procedure

The contents and wording of the questionnaire were pilot tested on a small number of chiropractors and then on a small number of students. After each testing, some wording and lay-out changes were made in response to this feedback to make the questionnaire more user-friendly.

Students in both chiropractic programs were informed of the nature of the project during class time or through distribution of an information brochure and that participation was voluntary and anonymous. The survey was distributed at the end of class time and students were informed of a designated return location. Ethics approval was granted by Murdoch University (Project No 2016/118) and was classed as negligible risk research.

The project followed the same protocols in both institutions, consent was obtained from students, data were non-identifiable (anonymous) and permission was obtained from the Head of the Macquarie University chiropractic program to conduct the research. Accordingly, the study met the criteria for classification under the National Statement on Ethical Conduct of Human Research (2007) (Sections 5.1.8 and 5.1.22) as exempt from requiring ethics approval from both Universities.

## Variables of interest and analysis of data

Data were entered and analysed in SPSS v.22 (IBM Corp, Armonk NY, USA) after having been cleaned. Survey items were dummy variable coded and descriptive statistics generated.

Outcome variables

1. Advice given to all patients - five health-related conditions
2. Excessive belief in chiropractic spinal adjustments – seven health-related conditions

The intention was to visually examine the responses of the participants on both research questions by tabulating them on a student year of study basis. Percentages were calculated for each of the responses to the two sets of questions and reported by year of study with their 95% confidence intervals (CI). These were shown in tables. We then sought to compare the responses between each year to see if there were any trends or if they differed. Consequently any differences in estimates between study years were identified and these were considered to be statistically significant if their 95% CIs did not overlap.

Preliminary analysis of the second set of questions ‘Belief in CSA’ revealed some of the “Definitely not” and “Yes, definitely” groups to be very small. Accordingly, we combined these responses with the “Probably not” and “Probably yes” groups respectively as we considered both types of responses to be indicative of disagreeing or agreeing.

Also, because of the small number of participants in the fifth year and the potential for that to impact on our results (particularly at MQ), we decided to conduct two analyses. In the first analyses, we combined the fourth and fifth year responses, assuming both years to be fairly similar in relation to their knowledge base on these health conditions. In the second analyses, the responses were calculated independently for each year, with the assumption that the final year students might change their opinions in view of their imminent entrance into the community as qualified health care professionals.

## Results

### Descriptive data

Out of a total of 831 possible participants in both programs, an overall response rate of 53% was obtained. As can be seen in Table 1, from a possible 313 Murdoch University chiropractic students, 216 (69%) chose to participate, while out of a possible 518 Macquarie University chiropractic students, 228 (44%) completed the surveys, giving a total of 444 students of whom 224 were males (50%). The two programs were combined in the analyses on the basis that there was no significant difference between the two programs on psychological and demographic variables. This has been reported elsewhere [12] .

**Table 1:** School, sex, and year of program

Year of Program	Males/Females	Response Rate by Year
	*n*	(%)
1st year MQ	43/34	**
MU	31/45	62%
2nd year MQ	17/10	**
MU	17/33	46%
3rd year MQ	42/20	**
MU	19/22	62%
4th year MQ	34/25	**
MU	6/21	79%
5th year MQ	3/0	**
MU	12/10	55%
All Years MQ	139/89	69%
MU	85/131	44%

** denotes information could not be provided because of the inability to ascertain students’ exact year of study

### Advice given to patients

#### Analyses with years 4 and 5 combined

Students in year 1 were found to have the lowest proportions of selecting ‘often / quite often’ of all the years for providing advice on all five ‘out of scope’ health conditions, whilst the students in years 4/ 5 had the highest proportions (see Table 2). There was no overlapping of the 95% CI for the ‘often, quite often’ response when comparing the estimates for the 1st year students to the combined estimates for 4th and 5th year students for ‘prevention of stress’, ‘cardiovascular disease’ and ‘diabetes’. This gradual increase was generally linear as the proportions tended to be incrementally higher in each successive year, the only exception being for musculoskeletal conditions. For students within their final 2 years of study, their lowest proportions were for the prevention of diabetes and cardiovascular disease, but these proportions were still higher than in the early years.

**Table 2:** Responses of Australian chiropractic students to the question of how often they would provide advice on a list of conditions in their future chiropractic practices reported separately for students in years 1 to 5, as well as in years 4 and 5 combined (“4/5”)

Question: In your practice will you give advice on …	*N*	Year	No or rarely	Sometimes	Quite often, Often
			*n* (%) [95% CI]	*n* (%) [95% CI]	*n* (%) [95% CI]
Prevention of stress:	153	1	10 (7) [3–12]	70 (46) [37–54]	73 (48) [40–56]
	77	2	5 (7)[2–15]	24 (31) [21–43]	48 (62) [51–73]
	103	3	5 (5) [2–11]	29 (28) [20–38]	69 (67) [57–76]
	86	4	0 (0) [0–4]	23 (27) [18–37]	63 (73) [63–82]
	25	5	1 (4) [1–20]	4 (16) [4–36]	20 (80) [59–93]
	111	4/5	1 (1) [0–5]	27 (24) [17–33]	83 (75) [67–83]
Prevention of cardiovascular disease	153	1	31 (20) [14–28]	62 (41) [33–49]	60 (39) [31–47]
	77	2	12 (16) [8–26]	35 (46) [34–57]	30 (39) [28–51]
	103	3	5 (5) [2–11]	37 (36) [27–46]	61 (59) [47–67]
	86	4	3 (4) [1–10]	18 (21) [13–31]	65 (76) [63–82]
	25	5	2 (8) [1–26]	6 (24) [9–45]	17 (68) [47–85]
	111	4/5	5 (5) [2–10]	24 (22) [14–30]	82 (74) [65–82]
Prevention of diabetes	153	1	41 (27) [20–35]	58 (38) [30–46]	54 (35) [28–43]
	77	2	13 (17) [9–27]	34 (44) [33–56]	30 (39) [28–51]
	103	3	6 (6) [2–12]	38 (37) [28–47]	59 (57) [47–67]
	86	4	2 (2) [0–8]	23 (27) [18–37]	61 (70) [60–80]
	25	5	1 (4) [1–20]	8 (32) [15–54]	16 (64) [43–82]
	111	4/5	3 (3) [1–8]	31 (28) [20–37]	77 (70) [60–78]
Prevention of musculoskeletal problems	153	1	0 (0) [0–2]	4 (3) [1–7]	149 (97) [93–99]
	77	2	1 (1) [0–7]	6 (8) [3–16]	60 (78) [67–87]
	103	3	0 (0) [0–3]	4 (4) [1–10]	99 (96) [90–99]
	86	4	0 (0) [0–4]	2 (2) [0–8]	84 (98) [92–100]
	25	5	0 (0) [0–14]	0 (0) [0–14]	25 (100) [86–100]
	111	4/5	0 (0) [0–3]	2 (2) [0–6]	109 (98) [94–99]
Wellness in general	153	1	1 (1) [0–4]	31 (20) [14–28]	121 (79) [72–85]
	77	2	1 (1) [0–7]	11 (14) [7–24]	65 (71) [74–91]
	103	3	0 (0) [0–3]	16 (16) [9–24]	87 (85) [76–91]
	86	4	0 (1) [0–6]	10 (12) [6–20]	75 (87) [78–93]
	25	5	0 (0) [0–14]	3 (12) [3–31]	22 (88) [69–98]
	111	4/5	1 (1) [0–5]	13 (12) [6–19]	97 (87) [78–94]

#### Years 1 through 5 analyses

As can be seen in Table 2, the 5th year students’ response proportions as compared to the earlier years did not follow the pattern of a gradual increase about giving advice on the health conditions of ‘cardiovascular disease’ and ‘diabetes’. For both conditions the proportions of students’ responses in years 1, 2, 3, and 4 gradually increased for ‘often / quite often’ and decreased for the response ‘sometimes’ and ‘no, rarely’. The 95% CI did not overlap for the 1st year students when compared to the 5th year students indicating that this difference was statistically significant. However, the 5th year students’ responses had a reverse pattern or remained constant when compared to the earlier years.

### Belief in chiropractic spinal adjustments

#### Analyses with years 4 and 5 combined

At least 7 out of every 10 chiropractic students in their final 2 years selected ‘yes, probably / definitely’ for the belief that CSA could prevent chronic back pain and help the body function at 100% of its capacity (see Table 3). Almost half of the years 4 and 5 students also selected this response for CSA being able to help the immune system, improve the health of infants, make it easier to give birth and prevent degeneration of the spine. Finally, almost one in five of the chiropractic students in these final years selected ‘Yes, probably / definitely’ that CSA could prevent disease in general.

**Table 3:** Opinions of chiropractic students on chiropractic spinal adjustments from years 1 through to 5, as well as the combination of years 4 and 5 (“4/5”)

Question: In your opinion, can chiropractic spinal adjustments	*n*	Year	Definitely not	Don’t know	Yes, probably
			Probably not		Yes, definitely
			*n* (%)[95% CI]	*n* (%)[95% CI]	*n* (%)[95% CI]
Prevent disease in general	153	1	64 (41) [34–50]	39 (26) [20–33]	50 (33) [25–41]
	77	2	31 (40) [29–52]	23 (30) [20–41]	23 (30) [20–41]
	103	3	41 (40) [30–50]	21 (20) [13–30]	41 (40) [30–50]
	86	4	55 (64) [53–74]	17 (20) [12–30]	14 (16) [9–26]
	25	5	10 (40) [21–63]	10 (40) [21–63]	5 (20) [7–41]
	111	4/5	65 (59) [49–68]	27 (24) [17–33]	19 (17) [11–25]
Prevent chronic back pain	153	1	2 (1) [0–5]	1 (1) [0–4]	150 (98) [94–99]
	77	2	1 (1) [0–7]	5 (7) [2–15]	71 (92) [84–97]
	103	3	2 (2) [0–7]	10 (10) [5–17]	90 (88) [79–93]
	86	4	5 (6) [2–13]	6 (7) [3–15]	75 (87) [78–93]
	25	5	1 (4) [0–20]	2 (8) [1–26]	22 (88) [69–98]
	111	4/5	6 (6) [2–11]	8 (7) [3–14]	97 (87) [78–93]
Help the immune system	153	1	30 (20) [13–27]	34 (22) [16–30]	88 (58) [49–66]
	77	2	12 (16) [8–26]	19 (24) [16–36]	46 (60) [48–71]
	103	3	24 (23) [16–33]	29 (28) [20–38]	50 (49) [39–59]
	86	4	27 (31) [22–42]	22 (26) [17–36]	37 (43) [32–54]
	25	5	2 (8) [1–25]	10 (40) [21–61]	13 (52) [31–72]
	111	4/5	29 (26) [18–35]	32 (29) [21–38]	50 (45) [36–55]
Make it easier to give birth	153	1	21 (13) [9–20]	57 (37) [30–45]	75 (49) [41–57]
	77	2	7 (9) [4–18]	24 (31) [21–43]	46 (60) [48–71]
	103	3	16 (16) [9–24]	33 (32) [23–42]	54 (52) [42–62]
	86	4	17 (20) [12–30]	31 (36) [26–47]	38 (44) [34–55]
	25	5	1 (4) [0–20]	8 (32) [15–54]	16 (64) [43–82]
	111	4/5	18 (16) [10–24]	39 (35) [26–54]	54 (48) [39–58]
Improve the health of infants	153	1	16 (11) [6–16]	46 (30) [23–38]	91 (60) [51–67]
	77	2	4 (5) [1–13]	23 (30) [20–41]	50 (65) [53–76]
	103	3	10 (10) [5–17]	37 (36) [27–46]	56 (55) [44–64]
	86	4	12 (14) [7–23]	28 (33) [23–44]	45 (53) [41–63]
	25	5	3 (12) [3–31]	6 (24) [9–45]	16 (64) [43–82]
	111	4/5	15 (14) [9–22]	34 (31) [26–45]	61 (54) [39–58]
Help the body function at 100% of its capacity	153	1	17 (11) [7–17]	18 (12) [7–18]	118 (77) [70–84]
	77	2	4 (5) [1–13]	10 (13) [6–23]	63 (82) [71–90]
	103	3	10 (10) [5–17]	15 (15) [8–23]	78 (76) [66–84]
	86	4	12 (14) [7–23]	15 (17) [10–27]	59 (69) [58–78]
	25	5	1 (4) [0–20]	3 (12) [3–31]	21 (84) [64–96]
	111	4/5	13 (10) [6–19]	18 (16) [10–24]	80 (72) [63–80]
Prevent degeneration of the spine	153	1	3 (2) [0–4]	14 (9) [5–15]	136 (89) [82–93]
	77	2	3 (4) [1–11]	8 (10) [5–19]	66 (86) [76–93]
	103	3	17 (17) [10–25]	15 (15) [8–23]	71 (69) [59–77]
	86	4	26 (30) [21–41]	16 (19) [11–28]	44 (51) [40–62]
	25	5	5 (20) [7–41]	3 (12) [3–31]	17 (68) [47–85]
	111	4/5	31 (28) [20–37]	19 (17) [11–25]	61 (55) [45–64]

At least 1 in 4 of these students in the final years of the program responded ‘Don’t know’ to the question can CSA ‘prevent disease in general’, ‘help the immune system’ and ‘make it easier to give birth’.

A lower proportion of students in their final years of study compared to students in the first year of study selected ‘Yes, probably / definitely’ that CSA could prevent ‘disease in general’, ‘prevent chronic back pain’, ‘help the immune system’, and ‘prevent degeneration of the spine’, respectively. The 95% CI did not overlap for ‘disease in general’, ‘prevent chronic back pain’ and ‘prevent degeneration of the spine’ indicating that this difference was statistically significant.

However, a higher proportion of students in their final years of study compared to students in the first year of study, believed that CSA could either ‘definitely not or probably not’ prevent or help any of the conditions except for ‘help the body function at 100%’. This was statistically significant for ‘prevent degeneration of the spine’.

#### Years 1 through 5 analyses

As can be seen in Table 3, the proportions of 5th year students who would respond ‘Definitely / probably not’ were consistently smaller than the proportions of the 4th year students (regardless of the question), while in contrast the proportions for the response ‘Definitely / probably yes’ were consistently larger. This reverses the general pattern of gradually decreasing levels of non-evidence-based beliefs in students up until the 5th year, when the pattern reverses and the proportions increase.

## Discussion

### Summary of findings

This is the first study to identify the frequency of non-evidence based beliefs in Australian chiropractic students. We also compared the proportions which hold these beliefs in each of the 5 years of the program. By the time they were ready for graduation, at least 2 in 3 of the chiropractic students saw themselves as sufficiently trained to advise patients ‘often’ on a range of non-musculoskeletal conditions. Final year students were approximately twice as likely to think this way as compared to first year students.

However, at least half of the chiropractic students also held non-evidence-based beliefs on the effects of CSA on 6 of the 7 health conditions that were illogical and unsupported with evidence. In general, the proportion of students in their final 2 years of the program, when compared to respondents from earlier years, were lower in these non-evidence-based beliefs. Nevertheless, the fifth-year students, specifically, demonstrated a reversal of the profiles observed in years 1 through to 4 for these beliefs. In other words, a larger proportion tended towards the more non-evidence based approaches in their final year.

### Discussion of findings and comparison with other studies

The responses of the chiropractic students in Australia and North America [29] were similar to each other for giving advice on non-musculoskeletal health conditions. The selection of ‘often’ indicates a belief that their future scope of practice will extend beyond musculoskeletal conditions. This seems unreasonable, as their undergraduate training emphasises musculoskeletal medicine. Conditions such as diabetes can be complex and challenging with serious consequences. Frequent advice at any greater depth without suitable training is ill-advised, unreasonable and outside the chiropractic students’ scope of practice. On the other hand, students may believe this is appropriate, as they receive training in dietetics, exercise and possibly stress reduction that will potentially have indirect benefits on diabetes or cardiovascular disease. Future student surveys could ask for greater detail as to what is understood by the word ‘advice’ with respect to each condition. This would clarify if the type of ‘advice’ is in accord with the level of training.

This study revealed a pattern of gradually increasing numbers of students who were prepared to give advice ‘often’ on all the health conditions. Perhaps with increasing knowledge or exposure to patients who are seeking advice a student becomes more aware of the potential through exercise and weight loss to impact on diabetes and cardiovascular disease. This could also be due to an ill-conceived bravado, overconfidence or a lack of clinical experience. What it does suggest is a developing belief that chiropractors are sufficiently educated to be able to provide clinically beneficial information outside the scope of musculoskeletal practice, a finding also similar to North American chiropractic students [30].

Another explanation has recently been suggested. Some forms of manual therapy (e.g., osteopathic treatment) have been shown to be associated with a professional identity based on a perception that its principles are unique, complex, and distinct [11]. This ‘philosophy’ is, by its proponents, deemed superior to science and appears to act as a cognitive lens through which practitioners and students view, judge and reject the results from research evidence and guidelines. This ‘lens’ effectively inverts the view of the traditional evidence pyramid and instead augments and elevates the value of personal experiences, anecdotes and the teachings of ‘expert’ therapists, whilst simultaneously diminishing and obscuring results from systematic reviews and meta-analyses. In our experience, this is often the case also in the chiropractic profession.

There was a suggestion that education might have some impact with the finding that 4th and 5th final year students’ proportions were lower for those who thought CSA could prevent or help with the various health conditions we had proposed, when compared to those proportions of the students from years 1, 2 and 3. However, these reductions were modest and over half of the students nearing graduation still thought that CSA could prevent degeneration of the spine and chronic back pain. Even more disconcerting was that nearly 1 in 5 of the 5th year students still thought CSA was preventative of disease in general.

Nevertheless, it was encouraging that the responses of the 5th year students in our study went against the pattern of gradually increasing numbers of students in each year of students being prepared to provide advice ‘often’ on cardiovascular disease and diabetes. In fact, numbers were gradually decreasing in sequential years for the number of students, who were adopting non-evidence-based beliefs for CSA, until 5th year when the numbers increased, reversing this pattern. This finding stands at odds with a curriculum which is intended to prepare them to be lifelong learners, and hence capable of delivering evidence-informed care. Implicit in our comments is the assumption that the curriculum is consistent across the years and does not change over time. On the other hand, one could hope that this result may be an artefact due to the small sample size in the fifth year. Nevertheless, it should not be disregarded, as it could be important information that provides researchers and educators with an educational time point that requires careful further investigation for contributing educational or environmental factors, such as the internship / clinical educational component.

Previous research with medical students has suggested that the transition from studentship to internship is often marked by a subjective sense of power [31]. Consequently, students become overconfident, which in turn has been associated with diagnostic errors and the selection of interventions that do not have an evidence base [32]. This may also be the case for chiropractic students, when they begin their clinical training and could account for the reversal patterns noted in the 5th year student responses in our study. Studies among medical practitioners, psychologists and nurses conflict as to whether accuracy improves with training, experience, reflection or feedback [32–34]. It is an interesting finding with considerable implications for chiropractic clinical educators and warrants further investigation.

In sum, it would appear that unsupported beliefs on the chiropractic scope of practice reduce to a modest degree over the course of chiropractic education but remain prevalent and resilient.

These findings are partly disconcerting and challenge the assumption that increasing knowledge extinguishes the likelihood of these non-evidence based beliefs. It raises several questions. How can chiropractic students express acceptable attitudes to guideline adherence and attitudes to functioning individuals with LBP [35], yet hold such unrealistic health beliefs? Is it possible that such discordant beliefs could be held also by other health care providers such as, medical practitioners, nurses or physiotherapists? Or is it only present in those with a strong professional identity underpinned with a “philosophy”? Are these irrational beliefs benign with no negative impact on guidelines based-care? Do they change with entry into the workforce? These are areas to be explored in the future and should include practicing chiropractors as well.

## Methodological considerations

Our two sets of questions were developed specifically to meet our needs and were pre-tested and refined. As they were fairly simple, we do not expect that students found them difficult to answer. There were almost no missing answers, which further strengthened our assumption that the questionnaire was user-friendly.

The response rate was relatively good for one chiropractic program but not so good for the other. Since the study was anonymous, we could not compare responders to non-responders. However, the profiles in relation to other factors were similar in the two programs and have been reported elsewhere [12]. We therefore assumed that the two student samples were similar. We combined the 4th and 5th year students to create a larger group because of the small number of 5th year student responders. However, as the result profiles were different in these two groups, we also separated them to report their data independently.

The questions asked for the ‘out of scope’ advice and health conditions were broad in nature. For example we could have asked “would you give advice to patients on their appropriate dosage of insulin” rather than “will you give advice on diabetes” or “does CSA prevent lung cancer”. As this paper was exploratory in nature we chose to begin with 5 common ‘big picture’ domains to gain an initial impression of student beliefs. A greater understanding of advice giving / ‘out of scope practice’ and unorthodox beliefs will be gained by more detailed questions in each of the domains.

Finally, the cross-sectional nature of this study limits any causal inferences that can be made about an individual’s knowledge progression over the course of chiropractic education. Hence, it would be relevant to follow the same cohort over its educational progression through the course. Intention to do something may not translate into reality, so it would also be relevant to follow them into practice to see if these intended advice patterns materialised.

## Conclusion

Evidence-based beliefs relating to musculoskeletal conditions are common in Australian chiropractic students but, at the same time, non-evidence-based beliefs are fairly common as well and are not dissimilar to those of chiropractic students in the North America. Australian students may not understand the limitations that education attempts to place on their scope of practice. Further, non-evidence-based beliefs appear to reduce somewhat but essentially remain resilient to change over the 5 years of their education. These findings suggest further research and a re-think on how chiropractic educators go about their business to produce graduates who understand and deliver evidence-based health care and are capable of integrating into the mainstream health care system.

## Additional file

Additional file 1: Anonymous Questionnaire for Chiropractic Students Survey. (DOCX 23 kb)

## Abbreviations

CSA: chiropractic spinal adjustments
LBP: Low back pain
MQ: Macquarie University
MSK: Musculoskeletal
MU: Murdoch University
NP: Neck pain
