# Supplementary material for: How frequent are non-evidence-based health care beliefs in chiropractic students and do they vary across the pre-professional educational years
Source: Chiropr Man Therap. 2018 Mar 15;26:8. doi: 10.1186/s12998-018-0178-y (PMC5853152; doi:10.1186/s12998-018-0178-y)
Supplement: Supplementary file 1 — Anonymous Questionnaire for Chiropractic Students Survey. (DOCX 23 kb) [file 12998_2018_178_MOESM1_ESM.docx]

**Appendix 1.**

**Anonymous Questionnaire for Chiropractic Students Survey**

**Participant consent**

I have read the Information letter and I agree to participate in this survey. Your answers are anonymous. The survey should take about 10 – 15 minutes to complete.

Thank you very much for your assistance.

Stanley Innes

Lecturer Chiropractic / Health Professions

Murdoch University

**Please answer the following questions**

Sex: Male  Female

Year of Program: Yr 1  Yr 2  Yr 3  Yr 4  Yr 5

No or rarely

*Sometimes*

*Quite often*

*Or often*

| 1. **In your practice will you give advice on** |  |  |  |
| --- | --- | --- | --- |
| prevention of stress |  |  |  |
| prevention of cardiovascular disease |  |  |  |
| prevention of diabetes |  |  |  |
| prevention of musculoskeletal problems |  |  |  |
| wellness in general |  |  |  |

*Don’t know*

*Definitely not*

*Probably not*

*Yes, probably*

*Yes, definitely*

| 1. **In your opinion, can chiropractic spinal adjustments** | | | | | |
| --- | --- | --- | --- | --- | --- |
| prevent disease in general? |  |  |  |  |  |
| prevent chronic back pain? |  |  |  |  |  |
| help the immune system? |  |  |  |  |  |
| make it easier to give birth? |  |  |  |  |  |
| improve the health of infants? |  |  |  |  |  |
| help the body function at 100% of its capacity? |  |  |  |  |  |
| prevent degeneration of the spine? |  |  |  |  |  |
